# Caffeine reduces deficits in mechanosensation and locomotion induced by L-DOPA and protects dopaminergic neurons in a transgenic *Caenorhabditis elegans* model of Parkinson’s disease

**DOI:** 10.1080/13880209.2020.1791192

**Published:** 2020-07-25

**Authors:** Rafael Vincent M. Manalo, Paul Mark B. Medina

**Affiliations:** Biological Models Laboratory, Department of Biochemistry and Molecular Biology, College of Medicine, University of the Philippines Manila, Manila, Philippines

**Keywords:** Sensation, neuroprotection, D2 receptors, reactive oxygen species

## Abstract

**Context:**

L-DOPA is the first-line drug for Parkinson’s disease (PD). However, chronic use can lead to dyskinesia. Caffeine, which is a known neuroprotectant, can potentially act as an adjunct to minimise adverse effects of L-DOPA.

**Objectives:**

This study determined changes in terms of neurodegeneration, locomotion and mechanosensation in *Caenorhabditis elegans* (Rhabditidae) strain UA57 overexpressing tyrosine hydroxylase (CAT-2) when treated with caffeine, L-DOPA or their combinations.

**Materials and methods:**

Neurodegeneration was monitored via fluorescence microscopy of GFP-tagged dopaminergic neurons in the head and tail regions of *C. elegans*. Meanwhile, mechanosensation and locomotion under vehicle (0.1% DMSO), L-DOPA (60 mM), caffeine (10 mM) or 60 mM L-DOPA + 10 or 20 mM caffeine (60LC10 and 60LC20) treatments were scored for 3 days (*n* = 20).

**Results:**

L-DOPA (60 mM) reduced CEP and ADE neurons by 4.3% on day 3, with a concomitant decrease in fluorescence by 44.6%. This correlated with reductions in gentle head (−35%) and nose touch (−40%) responses, but improved locomotion (20–75%) compared with vehicle alone. CEP and ADE neuron counts were preserved with caffeine (10 mM) or 60LC10 (98–100%), which correlated with improved mechanosensation (10–23%) and locomotion (18–76%). However, none of the treatments was able to preserve PDE neuron count, reducing the basal slowing response.

**Discussion and conclusions:**

Taken together, we show that caffeine can protect DAergic neurons and can reduce aberrant locomotion and loss of sensation when co-administered with L-DOPA, which can potentially impact PD treatment and warrants further investigation.

## Introduction

Parkinson’s disease (PD) affects 1–2% of the general population, usually the 65 years and above age-group, and is the second-most prevalent neurodegenerative disease globally (Alves et al. 2008; Liu et al. 2016). In the Philippines alone, 1% of Filipinos aged 50 and above have been diagnosed with PD, primarily of the idiopathic type (Gibrat et al. 2009). Certain pathological hallmarks are seen in PD, such as senescent or apoptotic dopaminergic neurons from the substantia nigra pars compacta, aggregation of α-synuclein proteins in Lewy bodies, progressive difficulty in motor control, and neurofibrillary tangles, to name a few (Gibrat et al. 2009; Weiner et al. 2013). What is striking is the fact that PD, as with other neurodegenerative disorders, has no approved treatment that can completely cure the disease.

Dopaminergic drugs, such as levodopa (L-DOPA) which is the gold standard, dopamine agonists, such as ropinirole, monoamine oxidase inhibitors and others are available for lessening the disease burden and increasing the patients’ quality of life. However, adverse motor events such as dyskinesia become more prevalent with long-term use. In the Philippine General Hospital, the prevalence of PD patients developing levodopa*-*induced dyskinesia (LID) was found to be at 36.11%, a significantly alarming proportion (Shiong-Shiu and Jamora 2015). Likewise, a study by Lee et al. (2011) showed that L-DOPA treatment to neuronal [SH-SY5Y] cells induced the aggregation of α-synuclein proteins, which aggravated in a dose-dependent manner. Altogether, these findings suggest that the quality of life of patients diagnosed with PD might have better prognoses with L-DOPA but may also progressively worsen whether or not they continue receiving the mainstay treatment.

Interestingly, new studies have been published regarding caffeine intake and the reduction in risk of developing PD, with pertinent data coming from patients in Asia such as Japan and Singapore (Abbas et al. 2018). For instance, a cohort study by Tan et al. (2008) showed that total caffeine intake by Singapore Chinese had an inverse relationship with PD development, garnering a relative risk of 0.55 or up to a 45% decrease in incidence. Likewise, several case-control studies have been done involving Japanese and Singaporeans that showed an odds ratio of 0.45–0.78 for PD development in patients taking caffeinated drinks (Costa et al. 2010; Tanaka et al. 2011). These clinical and epidemiological studies, apart from providing relevant data for the Asian population, have set a consistent pattern for caffeine and PD incidence. However, inasmuch as these studies promote caffeine intake for patients with PD, there is no mention of caffeine as an adjunct to L-DOPA, and evidence is still lacking on whether it can be used with the gold standard drug. Yet, caffeine remains to be a better candidate than other drugs for co-administration, due to its long-standing history of promising results.

Thus, current research aims to further understand how the adverse effects of L-DOPA could be reduced or minimised. Previously, we have shown that in a transgenic strain (UA57) of *Caenorhabditis elegans* (Rhabditidae), caffeine can protect dopaminergic (DA) neurons in the presence of dopamine overproduction (Manalo and Medina 2018a). This result suggested that caffeine can potentially work hand-in-hand with L-DOPA not only to prevent neurodegeneration but also to restore bodily function. In this study, we clarify the effects of caffeine by monitoring DA neurodegeneration as well as deficits in mechanosensation and locomotion, to determine if and how caffeine can overcome these losses.

## Materials and methods

The study conducted was divided into two phases: the first phase verified our previous findings on caffeine protection of dopaminergic neurons in UA57 (1–3 days). Briefly, five (5) treatment groups were used: (1) Negative control (0.1% DMSO) of the UA57 strain, (2) 60 mM L-DOPA, (3) 10 mM caffeine, (4) 60 mM L-DOPA + 10 mM caffeine, and (5) 60 mM L-DOPA + 20 mM caffeine. The concentration of caffeine (10 mM) was based on three studies: the highest mean lifespan extension of *C. elegans* at 20 °C with the greatest delay in age-related pathology using 10 mM caffeine (Sutphin et al. 2012); lifespan extension of *C. elegans* using 5 mM and 15 mM caffeine with significant developmental delay at 15 mM and reduced lifespan at 30 mM (Bridi et al. 2015), and our previous study showing persistent protection of DAergic neurons in *C. elegans* at 10 mM (Manalo and Medina 2018a). These therefore demonstrate that the safe and efficacious concentration of caffeine is at 10 mM, and an excessive concentration is within 15–30 mM caffeine, which we chose to be 20 mM. Meanwhile, the concentration of L-DOPA (60 mM) was initially based on a previous study showing improvement of dopaminergic behaviour in transgenic *C. elegans* using 20 mM L-DOPA (Yao et al. 2013). Since our current study explores the adverse effects of L-DOPA, we sought a concentration higher than 20 mM. This was therefore assessed by our laboratory using several concentrations of L-DOPA, and we found that the minimum concentration sufficient enough to induce deficits in sensation and locomotion in *C. elegans* was 60 mM (data not shown). For the vehicle, a previous study showed sir-2.1 and daf-16-dependent lifespan extension in *C. elegans* using 0.5% and 2% DMSO (Wang et al. 2010). To minimise the beneficial effects of DMSO to *C. elegans* which may confound the course of neurodegeneration of the control group, we used a dilution of 0.1%. This concentration is therefore safe as well as sufficient in obtaining proper dilutions of our treatment.

The second phase of the study monitored the phenotypic changes, primarily mechanosensation and locomotion, to correlate with the neuronal state of the worm. The same treatment groups were used, with the addition of wild-type strain N2 exposed to vehicle only (0.1% DMSO) to provide control data on the normal phenotype to which treatments were compared.

All work was done at the Biological Models Laboratory and the Cell Culture Laboratory of the Department of Biochemistry and Molecular Biology, College of Medicine, University of the Philippines Manila. The study was conducted at 20–25 °C and under the precautions of Biosafety Level 1. Further, the study was approved by the Research Ethics Board of the University of the Philippines Manila and was exempted from review due to the nature of the work, which involves the invertebrate animal model *C. elegans* (No. 2016-504-01).

### Preparation of nematode growth media

In this study, we used a transgenic strain of *C. elegans* overexpressing tyrosine hydroxylase CAT-2, which is a rate-limiting enzyme in dopamine biosynthesis (UA57). Hence, these nematodes overproduce dopamine to induce degeneration of DAergic neurons. *C. elegans* wild-type strain N2 was then used to serve as a phenotypic control for mechanosensation and locomotion assays. Nematodes were maintained in NGM plates prepared in the Biological Models Laboratory of UP Manila. Briefly, the following were weighed: 0.3 g NaCl, 0.25 g peptone and 2 g bacteriological agar, and dissolved in 100 mL of dH_2_O. The solution was autoclaved at 121 °C for 15 min, then cooled to 60 °C. After cooling, 100 µL each of the following was added: 1 M MgSO_4_, 1 M CaCl_2_, and 5 mg/mL cholesterol in 100% EtOH. Media were then poured into Petri plates using sterile techniques and stored at 4 °C. NGM plates were seeded with *E. coli* strain OP50 on the day of use.

### Age-synchronisation of* C. elegans*

Age synchronisation and sampling were based on the protocol of Manalo and Medina (2018a), with slight modifications. Briefly, nematodes (*N* = 15–20) at stage L4 to young-adulthood were transferred via worm-picking into prepared NGM plates seeded with *Escherichia coli* strain OP50 for all assays. In the fluorescence assay, the sample size for age-synchronized nematodes was *N* = 15 per treatment group due to time constraints in monitoring the dopaminergic neurons (four CEP, two ADE and two PDE neurons per worm, 15 worms per treatment group, five treatment groups). For all other assays (mechanosensation and locomotion), sample size was *N* = 20.

### Fluorescence microscopy of DA neurons

For this study, neurodegeneration was observed via fluorescence microscopy of GFP-tagged DAergic neurons in the head and tail regions of *C. elegans*. Briefly, nematodes were exposed to treatment and observed using the Evos^®^ FL inverted microscope for day 0. Succeeding observations were then made with the same worms for the cephalic (CEP), anterior deirid (ADE) and posterior deirid (PDE) neurons at days 1, 2 and 3 and analysed using Image J (US NIH). In the analysis, percent intact neurons out of the four CEP, two ADE and two PDE neurons were recorded per worm. Concomitantly, each neuron was traced and its GFP intensity was recorded. To analyse each intensity, the image was split into red, green, and blue channels by clicking Image → Colour → Split channels. For the whole analysis, only the green channel was used. To obtain the mean density of the background, freehand selection was used to randomly encircle the background thrice, with the mean density of the three measurements obtained. Each neuron was then encircled and the integrated density obtained. Lost neurons were scored with a GFP intensity of zero, while neurons too close to each other for delineation were traced as one and the resulting intensity was divided by the number of neurons encircled. The quotient was then scored for all neurons encircled. To quantify GFP intensity, the corrected total cell fluorescence (CTCF) of each neuron was obtained using the following formula:

(1) CTCF=Integrated density–(area delineated×mean density of background)

### Locomotion assay in* C. elegans*

Nematodes were exposed to the different treatments for a minimum of 2 h. Then, each worm was transferred to a blank NGM examination plate to minimise confounders due to external stimuli, and was allowed to roam for 1 min. This was based on the finding that worms exhibit an escape response for the first ∼100 s, and inhibits spontaneous reversals for up to 1 min after slight body touches, both of which may affect our observation (Zhao et al. 2003). Afterwards, each worm was recorded for 20 s, with the following behaviours quantified: short reversals, long reversals, total reversals, omega turns, and body bends (Zhao et al. 2003; Hart 2006). Each worm was then worm-picked back to separate OP50-seeded NGM plates for subsequent assays up to the 3^rd^ day. For all assays, response was recorded in absolute count (# response/20 s).

### Basal slowing response assay in* C. elegans*

This assay was patterned after the method of Rivard et al. (2010), with slight modifications. Briefly, the basal slowing response (BSR) was elicited by repeating the same method for observing body bending rate in the locomotion assay, but NGM examination plates were seeded with *E. coli* strain OP50. In this study, all OP50-seeded NGM plates were prepared fresh on each day of the examination to minimise changes in the plate texture as would occur when the bacterial lawn becomes thick and viscous, which might lead to a false-positive BSR. For body bending rate, response was recorded in absolute count (# response/20 s), after allowing each worm to roam for 1 min.

### Mechanosensation assay in* C. elegans*

For the mechanosensation assay, worm transfer to and from NGM examination plates was similar to the locomotion assay. For plate tap (during or after five subsequent plate tapping), gentle touch (head and tail), nose touch and harsh touch, the responses of the worms were recorded as a function of the total sample size (% response). For quantitation, a binary scoring system was used. Positive response was scored with 1, while a negative response was scored with 0. Parameters were on scoreable behaviours with expected responses in *C. elegans* previously published (Chalfie et al. 2014).

For gentle touch, a short and fine strand of hair was fixed on a sturdy base and used to strike the nose, head (pharynx area), and tail (anus area) – known as the nose touch, gentle touch (head) and gentle touch (tail) assays, respectively (Chalfie and Sulston 1981). The usual response of an adult nematode to plate tap, nose touch, and gentle touch (head) is a reversal (Chalfie and Sulston 1981; Kaplan and Horvitz 1993). Meanwhile, gentle touch (tail) will normally elicit a forward response. Each of the 20 animals was examined and the mean percentage scores obtained for each of the treatment groups.

For harsh touch, a nichrome wire was used to poke the midbody of each nematode. The usual response of the nematode to harsh touch is a reversal (Way and Chalfie 1989). In this study, we also counted forward movements only when an accelerated movement is evident, as this is also an indication of sensing midbody stimuli. Forward movements with no evident acceleration or a lack of change in direction were recorded as a negative response.

### Statistical analyses

Data were presented as mean ± standard error (S.E.) where absolute values are shown. To better compare percent responses with differing initial proportions, the adjusted response was computed for each parameter (as in mechanosensation). Briefly, the percent response of each day was adjusted relative to the response at day 0, and was presented as adjusted response ± %S.E. Data were compared using one-way analysis of variance (ANOVA) followed by a *post hoc* Bonferroni-holm method to correct for pairwise error rates due to multiple comparisons. Significance was labelled as “*” if *p* < 0.05 and “**” if *p* < 0.01.

## Results

**Figure 1.**
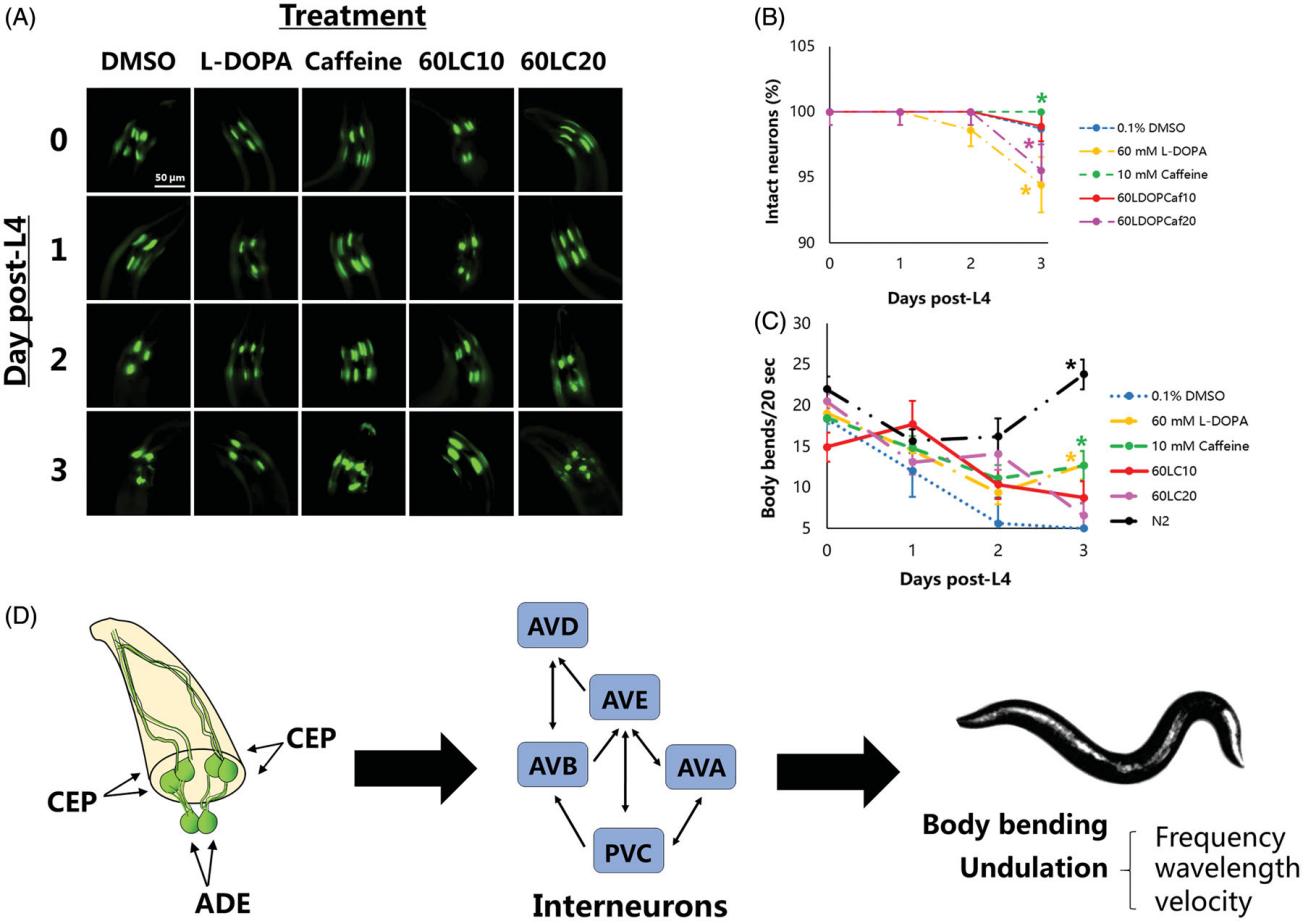
Caffeine or its combination with L-DOPA protects CEP and ADE neurons in transgenic *C. elegans*. Nematodes stage L4 to young adulthood were exposed to L-DOPA (60 mM) or caffeine (10 mM) and L-DOPA-caffeine combinations (60:10 mM and 60:20 mM) as compared to vehicle (0.1% DMSO). (A-B) DAergic neurodegeneration was worsened in the presence of L-DOPA alone (60 mM), which was prevented by co-administering caffeine (10 mM). (C) More numerous body bends were also observed in all treatment groups compared with vehicle. (D) The six DAergic neurons in the head region is hypothesised to consist the central pattern generator, which is responsible for basic movements and is important in maintaining proper locomotion. *p < 0.05 denotes significance. Treatment groups (mean ± S.E.) were compared via one-way ANOVA followed by a *post-hoc* Bonferroni-Holm method for multiple comparisons.

**Figure 2.**
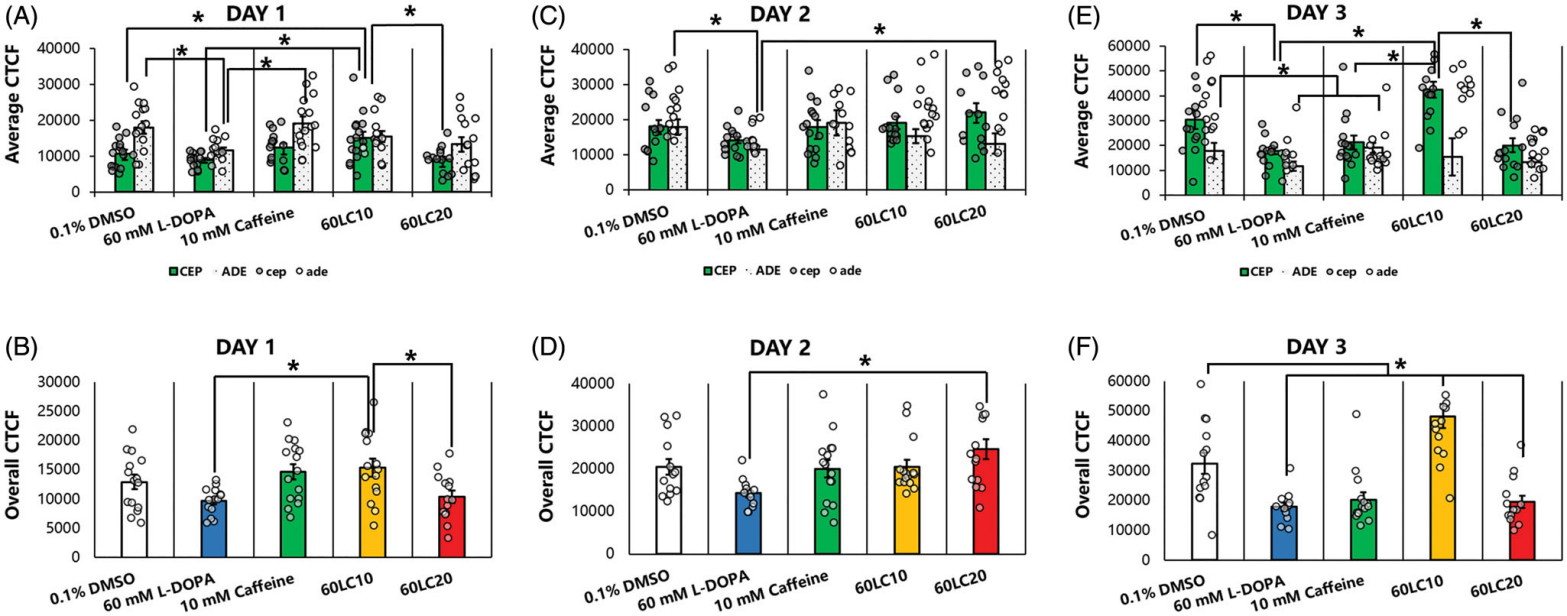
Caffeine or 60LC10 protects CEP and ADE dopaminergic neurons in *C. elegans*. (A,C,E) CTCF of the CEP and ADE neurons. Caffeine exposure leads to a stable GFP intensity from day 1 to day 3. Meanwhile, exposure to vehicle leads to increased intensity for CEP with concomitant decrease in intensity for ADE neurons. Exposure to 60LC10 drastically increases CEP intensity. Meanwhile, both the L-DOPA and 60LC20 groups showed progressively lower intensities. (B,D,F) Overall CTCF of the CEP and ADE neurons. *N* = 15. *p < 0.05 denotes significance. Treatment groups (mean ± S.E.) were compared via one-way ANOVA followed by a *post-hoc* Bonferroni-Holm method for multiple comparisons.

**Figure 3.**
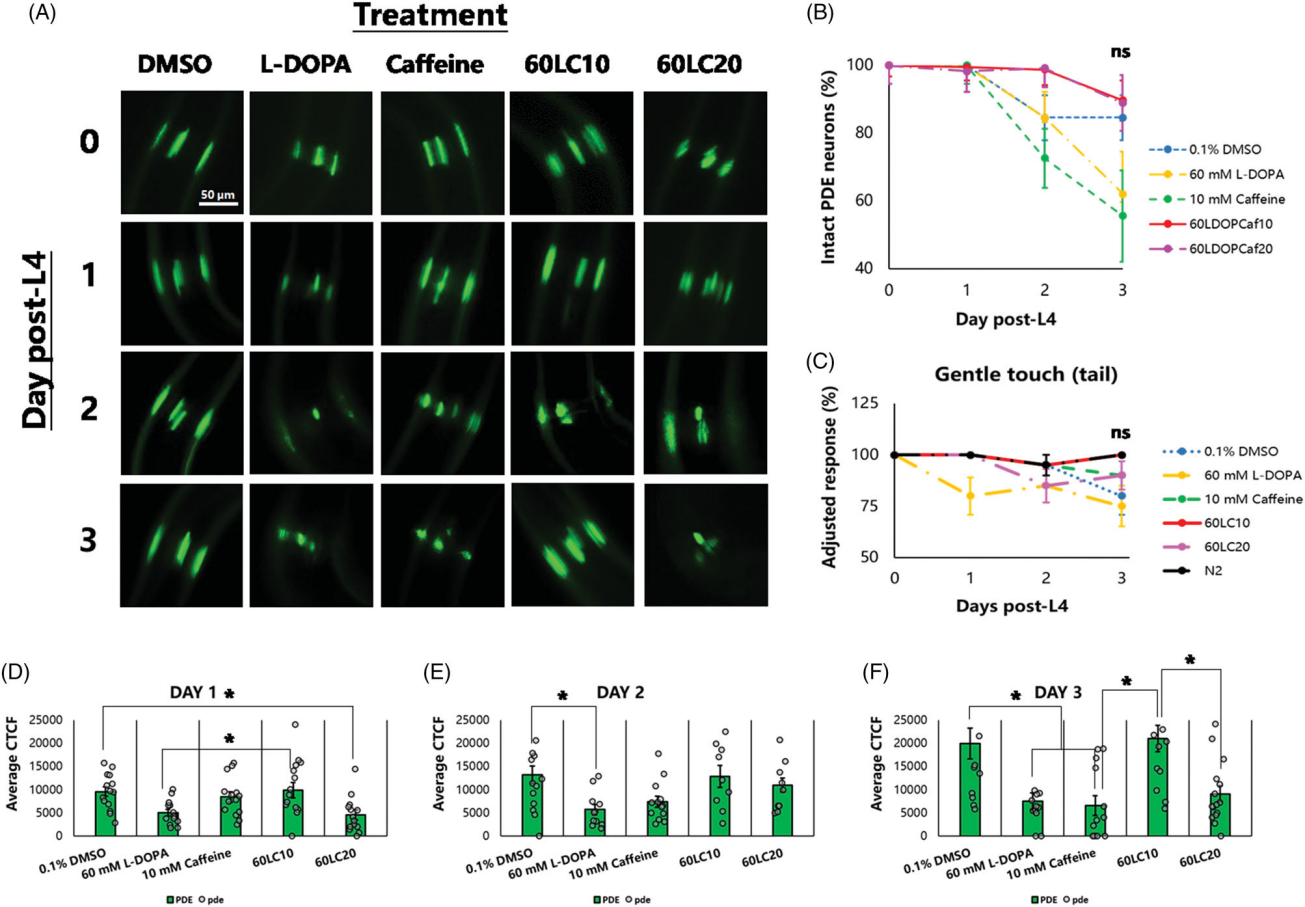
Caffeine or its combination with L-DOPA does not protect PDE neurons and leads to non-significant changes in tail touch response. Nematodes stage L4 to young adulthood were exposed to L-DOPA (60 mM) or caffeine (10 mM) and L-DOPA-caffeine combinations (60:10 mM and 60:20 mM) as compared to vehicle (0.1% DMSO). (A–B) Protection of DAergic PDE neurons was non-significant between vehicle and treatment groups. (C) This lack of protection correlated with a non-significant improvement in gentle tail sensation. (D–F) GFP intensity of the two PDE neurons were least in the caffeine (10 mM) and L-DOPA (60 mM) groups, suggesting detrimental effects. *p < 0.05 denotes significance. Treatment groups (mean ± S.E.) were compared via one-way ANOVA followed by a *post-hoc* Bonferroni-Holm method for multiple comparisons.

To evaluate the relationship of body-bending rate with neuroprotection, the six dopaminergic (DAergic) neurons in the anterior portion of *C. elegans* (namely, four cephalic and two anterior deirid neurons; CEP and ADE, respectively) as well as the two dopaminergic neurons in the posterior portion (namely, the posterior deirid neurons; PDE) were examined under different treatment groups and vehicle (0.1% DMSO). In previous reports, the six DA neurons in the head region of *C. elegans* were suggested to comprise the central pattern generator, a locus of synaptic connections responsible for nematode undulation frequency and velocity, based on laser ablation studies and their effects on locomotion (Rakowski et al. 2013). Hence, we sought to observe these neurons in this study, since undulation is a function of body bending (Figure 1(D)). Interestingly, all nematodes exposed to caffeine (10 mM), alone or in combination with L-DOPA (60 mM), showed six intact anterior DAergic neurons (Figure 1(A,B)) except when caffeine was in excess (20 mM). This finding was statistically significant compared with nematodes exposed to L-DOPA alone (60 mM) or to 60LC20 (*p* < 0.05). Meanwhile, lower GFP intensities of the ADE neurons compared to the CEP neurons were observed in all treatment groups except for caffeine (10 mM), with higher CEP intensities seen under exposure to vehicle or 60LC10 (Figure 2(A–E)). Meanwhile, caffeine showed relatively stable GFP intensities for each neuron type (CEP or ADE) and in total. This occurrence is not unusual, since the DAergic neurons that showed an increase in CEP intensity also showed a significant loss in ADE neurons. Hence, an increase in intensity may be indicative of compensation by the neurons remaining, a mechanism similar to a stress response (Manalo and Medina 2018b). In contrast, there was a non-significant decline in the percentage of intact PDE neurons (Figure 3(A–F)), which also correlated to a non-significant change in gentle touch to the tail between treatment groups and the phenotypic control (N2). However, a stark decrease in the GFP intensity was observed for both caffeine (10 mM) and L-DOPA (60 mM) when compared with vehicle, which was not observed when the two were co-administered (60LC10). The lack of protection by caffeine on the PDE neurons as suggested by the GFP intensities may be due to the lack of a circulatory system in *C. elegans* (Benian and Epstein 2011), which precludes effective drug distribution to the tail. In the context of CAT-2 overexpression in this strain (UA57) that leads to overproduction of endogenous L-DOPA, it seems that further elevation of neuronal L-DOPA by addition of an exogenous source (60 mM) worsens neurodegeneration but is still rescued by co-administration with caffeine (10 mM). In contrast, neuroprotection did not correlate with body-bending after further addition of L-DOPA (Figure 1(A,C)), since the L-DOPA treatment group showed the worst neuroprotection profile but was able to rescue body-bending rate significantly compared to vehicle (0.1% DMSO), which was also comparable to the caffeine treatment group (10 mM). This suggests that administration of exogenous L-DOPA can improve body-bending rate but does not prevent and in fact worsens neurodegeneration compared to control. This finding was found to be consistent with the current use of L-DOPA in improving movement in Parkinson’s disease and other hypokinetic movement disorders (Poewe et al. 2010; Varanese et al. 2011).

It is interesting to note that caffeine protected neurons from DA-induced neurodegeneration, which was consistent with our previous finding, and continues to do so even with further administration of exogenous L-DOPA (+60 mM). However, caffeine failed to exert its protection when its concentration was elevated further (20 mM), suggesting the existence of an overdose profile (Figure 1(B)). Further, body-bending rate significantly decreased when L-DOPA and caffeine (10 mM, 20 mM, shown as 60LC10 or 60LC20, respectively) were co-administered. This finding is independent of the neuronal profile, noting that nematodes exposed to 60LC20 had significant neurodegeneration compared with those exposed to 60LC10 (*p* < 0.05), yet the body-bending rates of both treatment groups were significantly less than that of caffeine or L-DOPA alone. Although these rates were significantly greater than in vehicle, these findings suggest that caffeine greatly reduces the rate of locomotion when co-administered with L-DOPA, which may point out to its effect on presynaptic autoreceptor dopamine D2-like receptors (DOP2Rs) that are inhibitory when dopamine release is in excess (Manalo and Medina 2018a).

**Figure 4.**
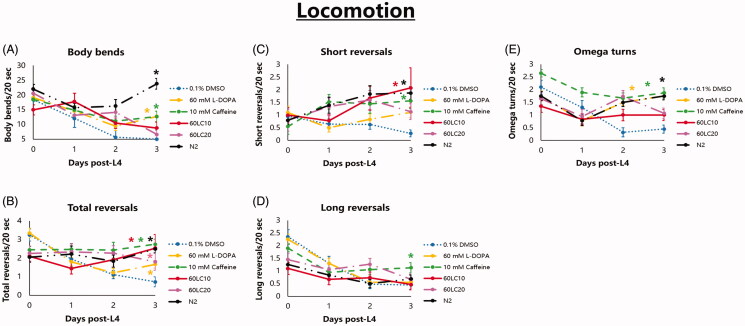
Locomotion profile of *C. elegans* UA57 is improved significantly by exposure to 60LC10 and is comparable to that of *C. elegans* strain N2. Nematodes stage L4 to young adulthood were exposed to L-DOPA (60 mM) or caffeine (10 mM) and L-DOPA-caffeine combinations (60LC10 or 60LC20) as compared to vehicle or N2 (0.1% DMSO). (A) Body bending rate is significantly improved by L-DOPA, an effect that is expected from the pharmacodynamics of the drug. However, neither L-DOPA nor caffeine can restore body bending back to normal. (B–D) Exposure to L-DOPA, caffeine, or their combinations (60LC10 or 60LC20) significantly improves total reversals, with greater contribution coming from short reversals. Meanwhile, only the caffeine group can increase long reversals in the worm. (E) Omega turning, which is a complex function *C. elegans* uses to abruptly change bearing, is preserved by exposure to L-DOPA or caffeine. *N* = 20. *p < 0.05 denotes significance. Rate-time (mean ± S.E.) data was analysed via one-way ANOVA followed by a *post-hoc* Bonferroni-Holm method for multiple comparisons.

**Figure 5.**
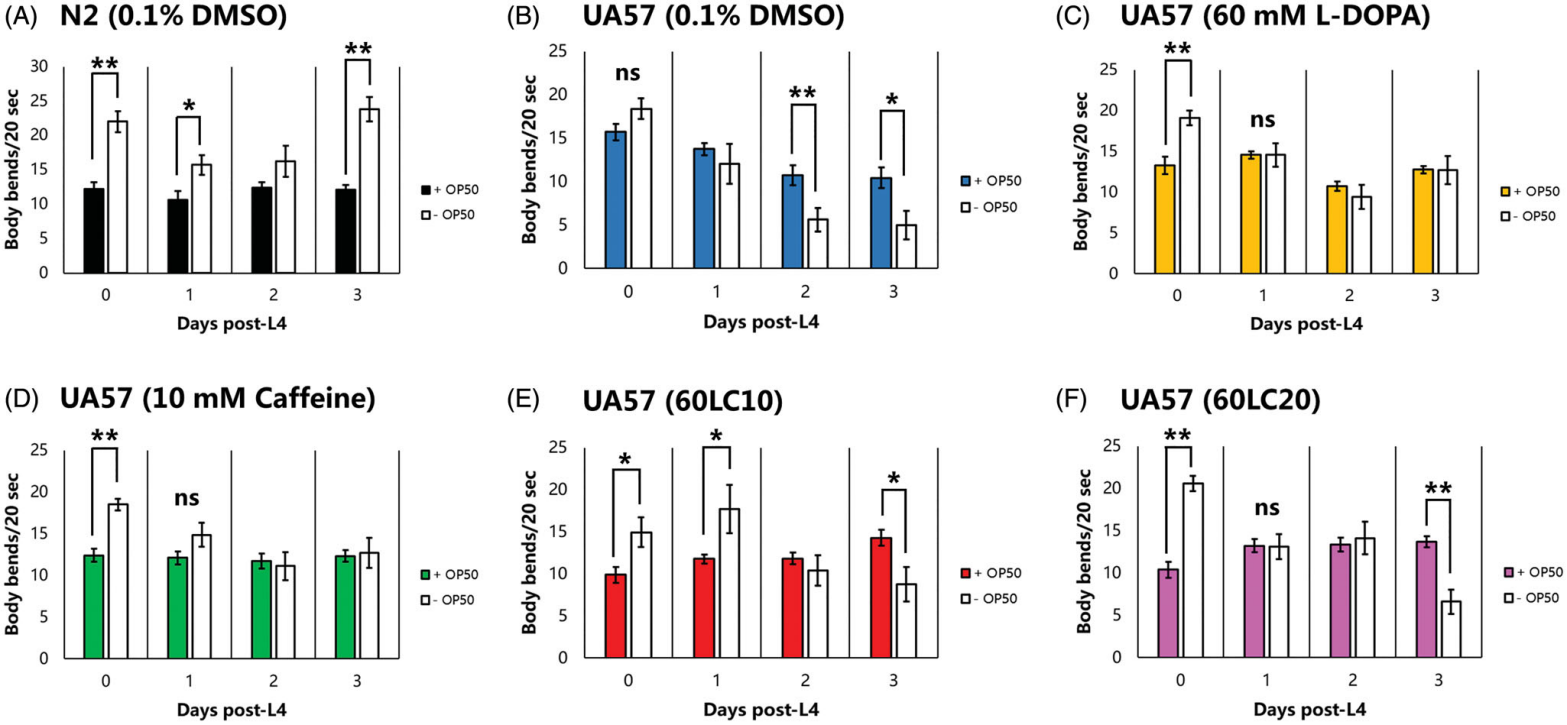
Caffeine or its combination with L-DOPA does not protect the basal slowing response. Nematodes stage L4 to young adulthood were exposed to L-DOPA (60 mM) or caffeine (10 mM) and L-DOPA-caffeine combinations (60LC10 or 60LC20) as compared to vehicle or N2 (0.1% DMSO). (A) Wild-type *C. elegans* (N2) showed persistent basal slowing response with reductions in body bending rate ranging from 24% to 49%. *p < 0.05 and **p < 0.01 denote significance. Rate-time (mean ± S.E.) data was analysed via one-way ANOVA followed by a *post-hoc* Bonferroni-Holm method for multiple comparisons.

In terms of locomotion, nematodes (UA57) made significantly more body bends when exposed to L-DOPA (60 mM) and caffeine (10 mM), compared with both vehicle (0.1% DMSO) and L-DOPA:caffeine co-administrations (Figure 4(A)) on the 3rd day. Meanwhile, total reversals were lessened when nematodes were exposed to L-DOPA (60 mM), or when L-DOPA was co-administered with an overdose of caffeine (20 mM). When nematodes were exposed to caffeine only (10 mM), the total number of reversals increased significantly (Figure 4(B)), which was also five-fold higher than in vehicle (0.1% DMSO). Further, caffeine at the said concentration (10 mM) was able to maintain the reversal rate even when co-administered with 60 mM L-DOPA, suggesting a protective effect on locomotion. In a span of three days, long reversals were relatively maintained from the 1st to the 3rd day, while short reversals increased, resulting to a net increase in total reversals for nematodes exposed to 10 mM caffeine (Figure 4(B–D)). This observation, interestingly, was found to be similar with the 60LC10 group. The combination of preserved locomotion and improved total reversals suggests that 10 mM caffeine or 60LC10 improves locomotion rate in *C. elegans* and can improve the rate of total reversals and omega turns comparable to those of strain N2, with the sole exception of body bending. To verify the results suggestive of protection of dopaminergic function, we also monitored the body bending rate of both wild-type (N2) and transgenic (UA57) *C. elegans* in the presence of freshly-prepared OP50-seeded NGM plates to record the presence or absence of the basal slowing response. Results showed that wild-type nematodes had preserved basal slowing responses for the span of the assay (3 days), with slowing rates ranging from 24% to 49% (Figure 5). Meanwhile, no treatment was able to preserve the basal slowing response on the 3rd day (Figure 5(E)). The loss of basal slowing response despite protection of most anterior DAergic neurons (CEP, ADE) suggests that PDE neurons have a greater influence on the basal slowing response than do the other six neurons.

**Figure 6.**
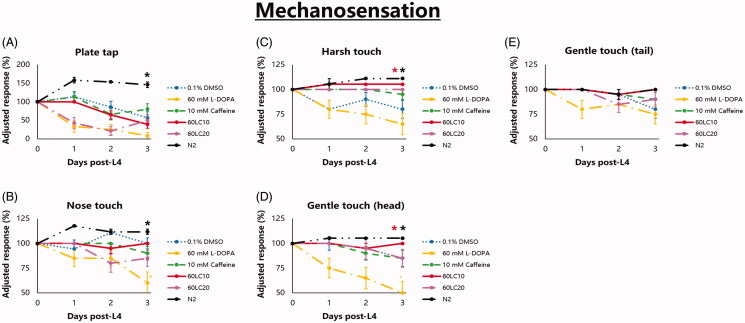
Mechanosensation profile of *C. elegans* UA57 is improved significantly by exposure to 60LC10 and is comparable to that of *C. elegans* strain N2. Nematodes stage L4 to young adulthood were exposed to L-DOPA (60 mM) or caffeine (10 mM) and L-DOPA-caffeine combinations (60LC10 or 60LC20) as compared to vehicle or N2 (0.1% DMSO). (A) General sensation declined within 3 days, with notably faster decline in the vehicle and L-DOPA groups. (B–D) In the nose and harsh touches as well as gentle touch to the head, exogenous L-DOPA resulted to the greatest decline in responses. Meanwhile, 60LC10 rescued these sensations back to the normal phenotype (N2), except for nose touch. (E) Response to gentle touch (tail) is not significantly affected by any treatment group. *N* = 20. *p < 0.05 denotes significance. Rate-time (mean ± S.E.) data was analysed via one-way ANOVA followed by a *post-hoc* Bonferroni-Holm method for multiple comparisons.

To further evaluate the effects of caffeine and L-DOPA on behaviour, the state of mechanosensation was observed in *C. elegans* (Figure 6). This is due to a previous study reporting numbness in about 38% of PD patients, which was then supported years later by another study correlating idiopathic PD with peripheral neuropathy (Koller 1984; Podgorny and Toth, 2014). Based on our observation, the normal profile of the UA57 strain exposed to only to vehicle was a gradual decline in specific sensations (nose touch, harsh touch, light touch) and a swift decline in general sensation as indicated by the plate tap response (Figure 4(A)). Meanwhile, addition of exogenous L-DOPA (60 mM) speeds up the decline of both types of sensations, noting an almost complete loss of response to plate tap (Figure 4(A-E)). Caffeine exposure (10 mM) significantly retarded decline in sensation, specifically harsh touch and gentle touch, only when in combination with L-DOPA (60LC10), and lost this protective effect at twice the concentration (20 mM). Further, although insignificant (*p* < 0.05), caffeine at 10 mM preserved nose touch response when compared with 60 mM L-DOPA (Figure 6(B)). Generally, the lack of significance in the mechanosensation assay may have been due to the binary approach of the scoring response, where 1 and 0 truly have a large difference that can affect standard errors. Hence, it is recommended that more precise scoring systems be devised in future studies. Nonetheless, even under a stringent scoring system, 60LC10 was able to protect harsh touch and gentle touch to the head and to rescue it back to the normal phenotype of strain N2 (Figure 6(A)). This suggests a notable effect of caffeine on sensation when co-administered with L-DOPA at an optimal concentration.

## Discussion

**Figure 7.**
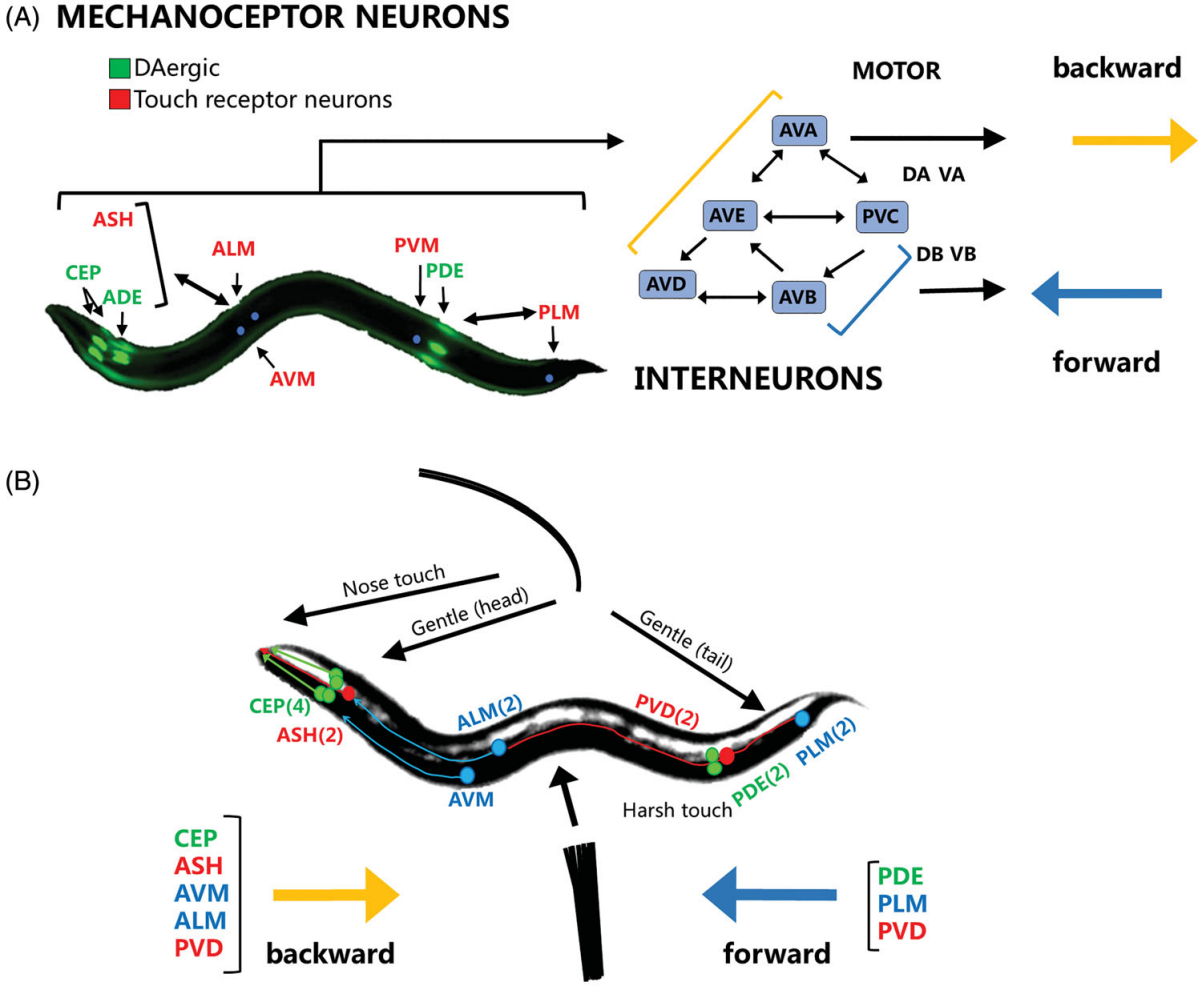
Connections between dopaminergic and touch receptor neurons in eliciting spontaneous forward or backward locomotion or responses based on mechanosensory input in *C. elegans*. (A) DAergic and touch receptor neurons, which make up the mechanoceptor system, coordinates with interneurons postulated to be the central pattern generator, which then relay the signal to motor neurons to elicit a forward or backward movement, or a combination of both to create complex movements. (B) Nematodes display a well-elucidated neural pathway for mechanosensation, the output of which may be discriminated and elicited using a hair brush and a nichrome wire for light and hard touches, respectively. A reversal is usually the result of communication between CEP dopaminergic (DAergic) and ASH, AVM, ALM, and PVD touch receptor neurons. Meanwhile, forward locomotion is usually elicited by the PLM and PVD touch receptor neurons, often in communication with the PDE DAergic neurons.

Previously, we have shown that *C. elegans* strain UA57 exposed to 10 mM caffeine had less degeneration of the four cephalic and two anterior deirid neurons, which otherwise would have degenerated drastically in vehicle (0.1% DMSO). Further, we elucidated and proposed a mechanism thought to be conserved between *C. elegans* and mammals, that is, the close communication between adenosine receptor antagonism and activation of dopamine D2-like receptors (DOP2Rs) in modulating excessive dopamine synthesis or release (Manalo and Medina 2018a). However, there is no clear-cut correlation between the neuroprotection conferred by caffeine and its effects on behaviour, which is probably more relevant to drug discovery and development for Parkinson’s disease. Recently, laser ablation and computational modelling studies suggested a collective role for interneurons in communicating as a central pattern generator (CPG), a neural network conserved among species that determines the pattern of motor action. In humans, the CPG is important for breathing, walking and swimming, among others. In *C. elegans,* the CPG is thought to mediate body bending, undulation amplitude and frequency (Poewe et al. 2010; Gjorgjieva et al. 2014). Meanwhile, DAergic neurons – which communicate closely with the interneurons (Figures 1(D) and 7(A,B)), mediate behaviours such as local searching, mechanosensation, gait switching, and decision-making (Vidal-Gadea and Pierce-Shimomura 2012). A study by Wei Li et al. (2006) showed that TRP-4 acts on a single neuron, DVA, to mediate proprioception and fine-tune motor activity. Interestingly, at least two other studies have shown that the trp-4 gene is expressed in CEP and ADE neurons (Li et al. 2006; Kang et al. 2010), suggesting that CEP and ADE neurons are essential for body bending as they relay the initiating signal to DVA. Meanwhile, at least three studies have shown a clear-cut correlation between the dopaminergic pathway and *C. elegans* reversals: (1) First, dopamine signalling has been shown to activate the anterior mechanoceptor neurons to fine-tune reversal or to accelerate tap escape (Kindt et al. 2007); (2) omega turning has been shown to increase with increasing long reversals, the latter of which is mediated by DAergic neurons (Zhao et al. 2003), and (3) synaptic input from the touch neurons (ALM, PLM) can control dopamine release from CEP and PDE neurons to act as a feedback loop in modulating touch responses (Sanyal et al. 2004).

Hence, any aberrations in dopamine signalling can lead to observable changes in locomotion and mechanosensation in *C. elegans.* This phenomenon is similar to gait dysfunction, tremors, rigidity and bradykinesia as seen in Parkinson’s disease. Thus, the use of the *C. elegans* model to determine the effects of caffeine on dopamine signalling and behaviour is possible and relevant, given its advantage of speed, translucency of body lining and conservation of genes and neural pathways.

In this study, the transgenic *C. elegans* strain UA57 overexpressed CAT-2, leading to overproduction of endogenous L-DOPA and hence dopamine in DAergic neurons. To this end, exposure of worms to caffeine alone does not suggest protection in isolation; rather, results are suggestive of caffeine exposure when excess dopamine is already present. In neurons, excess dopamine can produce the following relevant mechanisms: (1) overproduction of reactive oxygen species from its synthesis and degradation, (2) excessive Ca^2+^ influx into the cell to depolarise the membrane and promote vesicle release, and (3) rapid turnover of enzymes due to increased workload. These three mechanisms can, in part, provide bases as to how CAT-2 overexpression can lead to neurodegeneration. Co-administration of exogenous L-DOPA with caffeine (60LC10 or 60LC20) therefore looks at the limit of caffeine protection when further challenged by more dopamine, rather than reflecting the effects of caffeine and L-DOPA when given together to a healthy animal. Results of this approach are important in predicting the effects of caffeine in late-stage PD, when administration of L-DOPA is substantially high and neuronal state is skewed towards neurodegeneration.

Regarding the GFP fluorescence intensity of neurons exposed to vehicle, we hypothesise that the paradoxical increase despite degeneration is due to the compensation of CEP neurons for the damage or loss of other neurons such as ADE. An increase in function, therefore, can result in greater expression of proteins and can lead either to neuron growth or merely an increase in GFP expression. In the 60LC10 group, we hypothesise that caffeine at 10 mM is able to promote survival of all neurons despite an additional stressor (60 mM L-DOPA), allowing all neurons to survive but resulting to an increase in GFP proportional to the amount of stress, possibly pointing to the ER stress response (Manalo and Medina 2018b). A newly discovered mechanism involving mitochondrial dysfunction shows that ROS can increase hop-1 and pink-1 expression in *C. elegans,* which can lead to motor deficits and neurodegeneration. Further, since Ca^2+^ influx to the cell is excessive when dopamine is overproduced, a danger arises regarding Ca^2+^ overloading of the ER, which can lead to further mitochondrial dysfunction if the integrity of the TMCO1 channel is compromised by prior ROS-induced damage. Hence, it is possible that caffeine protects DAergic neurons by attenuating hop-1 or pink-1 expression and reducing intracellular ROS effects to preserve an ER TMCO1 channel ortholog in *C. elegans*, ultimately protecting the neurons in the presence of excessive dopamine (Wu et al. 2018; Wang et al. 2019). In contrast, we hypothesise that the lack of efficient caffeine delivery to the tail resulted in aberrant effects that may have been detrimental to the PDE neurons (Figure 3). This resulting subpar tail concentration of caffeine can lead to less protection against oxidative stress, as afforded directly by caffeine, while still activating presynaptic DOP2Rs, which may result to neurodegeneration from cytosolic dopamine accumulation (Manalo 2019), explaining the loss of GFP intensity to the PDE neurons. When co-administered with L-DOPA, the amount of dopamine produced by each PDE neuron may increase, allowing stable dopamine release that may reduce the compensation stress on each neuron (Figure 3(F)).

In terms of phenotypic changes, we establish that the chronic presence of excessive L-DOPA in DA neurons leads to a time-dependent reduction in locomotion and mechanosensation (Figures 4(A) and 6(A)). Intuitively, we would expect that body bending, which is mediated by CEP, ADE, and PDE neurons, would be worsened in worms further exposed to 60 mM L-DOPA; yet, the contrary was true. In worms exposed to exogenous L-DOPA, body bending was significantly higher than in control (0.1% DMSO) and was comparable to the caffeine treatment group (10 mM) on day 3. This confirms the conserved effect of L-DOPA in the *C. elegans* model. In humans, L-DOPA has been shown to improve locomotion of patients with PD but can also precipitate psychosis and dyskinesias at persistently high doses (Poewe et al. 2010; Varanese et al. 2011). Further, high-dose L-DOPA has been shown to promote neuronal cell death through oxidative and non-oxidative mechanisms *in vitro* (Pedrosa and Soares-da-Silva 2002). *In vivo*, evidence is scarce, but dopaminergic function declines more rapidly in patients receiving L-DOPA, and pathological studies, albeit cross-sectional, has demonstrated evidence of accelerated loss of DAergic neurons (Olanow 2015). Yet, L-DOPA can remain efficacious and is still recommended in late-stage PD (Varanese et al. 2011). Hence, the observation that L-DOPA worsens neurodegeneration yet improves body bending in the *C. elegans* model is in fact not uncommon.

Further analysis of the locomotion phenotype revealed that 60 mM L-DOPA can improve short, long, and total reversals as well as omega turns over time when compared with vehicle (*p* < 0.05). However, locomotion still continued to decline (Figure 4), suggesting that the decrease of locomotion in the vehicle group was a function of both tolerance and neurodegeneration. Ergo, exposure to 60 mM L-DOPA recovers the motor response, before the worm can grow tolerant once again. A previous study reported hyperactivity with lack of purpose in L-DOPA-treated *C. elegans* (Gupta et al. 2016), which was consistent with our study showing greater locomotion scores but with increasing neurodegeneration. the latter being the probable reason for the loss in prudent locomotion. Caffeine, on the other hand, was able to preserve locomotion parameters throughout the assay time, with initial decline seen in long reversals and omega turns but resulting to an eventual plateau. To explain the relevance of these results, it has been previously shown that the nematode *C. elegans* can perform a meaningful reversal to change its bearing, often ending in an omega turn if the reversal is a long reversal (Zhao et al. 2003). Hence, the greater number of long reversals due to caffeine exposure can lead to greater omega turns. This has implications regarding the execution of complex, meaningful movements. Through this simple model, we show insights on how caffeine may promote purposive locomotion.

Both L-DOPA:caffeine mixtures (60LC10 and 60LC20) resulted to improved locomotion compared with vehicle (0.1% DMSO), but lower than when only caffeine (10 mM) was administered (Figure 4). In a similar context, this shows that caffeine can protect locomotion only at a certain level of high-dose of L-DOPA, and that further increases (+60 mM) in L-DOPA can reduce caffeine benefit. Further, these results show that caffeine benefit at very high doses of L-DOPA cannot be rescued by increasing caffeine concentration (20 mM), because caffeine excess may have caused toxicity.

Meanwhile, we sought to correlate our findings with basal slowing response and GFP intensities, where we found a lack of slowing behaviour on the 3rd day and the lack of significant protection of PDE neurons by any of the treatment groups. Previously, it was shown that single ablation of the four CEP neurons led to only a modest defect in the BSR, while double ablation of the four CEP and two ADE neurons did not affect the BSR. Meanwhile, ablation of the four CEP and two PDE neurons significantly affected the BSR (Sawin et al. 2000). These findings suggest that the effects of the eight DAergic neurons are redundant, and that the combined functions of CEP and PDE neuron types work to preserve the BSR. In our results showing the CTCF of the six anterior DAergic neurons, only 60LC10 was able to significantly protect CEP neurons on the 3rd day, compared with vehicle (Figure 2(E)). This may in fact support the finding that 60LC10 was the only treatment that preserved the basal slowing response until the 1st day, because observable degeneration in both CEP and PDE neurons can intuitively affect the slowing response.

Similarly, we found that general sensation (as shown by plate tap) declined the fastest (Figure 6(A)). The plate tap response activates all touch receptor neurons, which is seen as a combined activation of anterior touch (ALM, AVM) and posterior touch (PLM) neurons (Wicks and Rankin 1995). In an adult worm, the anterior touch receptor neurons dominate and the worms typically exhibit a reversal (Chalfie et al. 1985; Wicks et al. 1996; Goodman 2006). In our results, we postulate that aside from the touch receptor neurons, the AVE and AVA interneurons provide greater stimulation to the DA and VA motor neurons (Riddle et al. 1997), allowing the nematode to elicit a backward response or reversal (Figure 7). Hence, the lack of response in the plate tap assay suggests either an increase in stimulation from neurons promoting a forward response, or a decrease in stimulation from neurons promoting a backward response, with the latter being more plausible. Of the dopaminergic and touch receptor neurons, the CEP DAergic and ASH, AVM, ALM, and PVD touch receptor neurons elicit a backward response (Figure 7(A,B)), while the PDE DAergic and PLM and PVD touch receptor neurons mediate forward locomotion (Riddle et al. 1997; Pokala et al. 2014; Yan et al. 2017). Hence, based on the fluorescence images showing DAergic neurodegeneration, it is likely that losses in CEP neurons accounted for the swift decline in plate tap response. Loss of response is not readily evident in other specific sensations because the plate tap checks the competing stimulation of ‘forward’ and ‘backward’ neurons, while specific sensation assays (light touch, nose touch, hard touch) check if the forward or backward response is still intact. Hence, a worm may have an intact backward response, but may not reverse in the plate tap because the backward stimulation has been overpowered by the forward stimulation. In a healthy, adult worm, the backward stimulation is usually stronger.

For the more rapid decline in plate tap response seen among L-DOPA treated nematodes, it is likely that caffeine preserves the strength of the backward response, presumably by protecting the CEP DAergic neurons. This suggestion is in fact supported by the constant preservation of DAergic neurons as well as the maintenance of the CEP CTCF in caffeine-exposed worms, which implied constant protection from stress and a lack of compensation. Meanwhile, all specific sensations, except for the tail response, showed a slower decline in all caffeine or 60LC10-treated worms, with the exception of worms exposed to excessive caffeine (20 mM). This suggests that caffeine preserves more anterior than posterior DAergic neurons, except when given in overdose (Figures 1(A) and 3(A)). Further, the notably refractory nature of the tail response to any caffeine-L-DOPA combination suggests that one or more neurons in the tail region of *C. elegans* (PVM, PDE, PLM) are not protected. The true effects of caffeine on these neurons is difficult to ascertain, given the distance of the tail region from the head and intestine which may prevent it from directly benefitting from caffeine, if the diffusive nature of its absorption is considered (Hulme and Whitesides 2011). Notably, 60LC10 can rescue responses to harsh touch and gentle touch to the head back to the normal phenotype, as shown in Figure 6, with all others being insignificant. These suggest that although caffeine protects both forward and backward neurons, it cannot completely protect ‘backward’ neurons from degeneration. On the other hand, it could also be due to the binary approach of scoring, which as aforementioned may have masked modest effects. The relevance of these findings points to the competing nature of stimuli. In reality, different stimuli send signals to the body all at the same time, and neural networks then play a role in reading messages from the external environment and providing an action based on the given condition. Hence, caffeine may preserve specific sensations when used singly, but may not be able to restore complex discrimination when various stimuli are sensed.

To further explain why caffeine could not rescue plate tap response and response to gentle touch (head) as in wild-type, consider that caffeine exposure protected CEP and ADE neurons over time relative to vehicle (Figures 1(A) and 2(E)). Further, in the harsh touch assay, 60LC10 exposure led to a backward response comparable to that in N2. This suggests that CEP and PVD neurons are protected, and their state cannot therefore explain the sub-optimal plate tap response in UA57, supposedly due to a weaker backward signal. However, in the nose touch assay which directly monitored ASH neurons, caffeine exposure was notably insignificant compared with vehicle (Figure 6(B)). Hence, it is possible that caffeine cannot rescue the plate tap response because it poorly protects the ASH neuron. Further investigation on this, as well as on the AVM and ALM touch receptor neurons, is warranted.

For body bending, we postulate a role in our proposed mechanism involving adenosine receptors and DOP2Rs. Since DOP2Rs are auto-receptors that modulate dopamine synthesis or release upon reaching a threshold (Ford 2014; Robinson et al. 2017), it seems logical that exposure to caffeine would lower this threshold in lieu of its activation of DOP2Rs, such that it may protect DA neurons but at the expense of body bending. Other parameters on locomotion such as the rates of reversals and omega turns, on the other hand, were comparable to wild-type probably because they are motions of need, which the nematode executes only at certain times. This is in contrast to body bending, which occurs all throughout the movement of the worm as a derivative of its undulation. When L-DOPA is added, the threshold is easily reached, so that even 60LC10 cannot rescue body bending back to the normal phenotype. However, since DA neurons are evidently protected, the losses of movement and sensation are much less or even not observable – providing behavioural advantage to the pathologic *C. elegans.*

## Conclusion

In this study, we show that caffeine at a concentration of 10 mM, alone or in combination with exogenous L-DOPA (60 mM), can protect DA neurons from neurodegeneration under intrinsic dopamine excess. Further, exposure to caffeine (10 mM) or its combination with L-DOPA (60LC10) can reduce deficits in certain parameters of mechanosensation and locomotion, namely: harsh touch and gentle touch to the head for sensation and body bending, reversals (short, long and total) and omega turns for mechanosensation and locomotion, respectively. Further, these behaviours are enhanced to a level comparable with those of a wild-type N2 strain, except for body bending. Similarly, none of the treatment groups was able to preserve the basal slowing response of body bending, in contrast to the wild-type N2 strain. Lastly, caffeine given at double the concentration (20 mM) showed reduced protection at several phenotypic parameters. This suggests that caffeine might have an overdose profile, whose effects may be conserved in the *C. elegans* model. Overall, these results suggest that caffeine can be an efficacious adjunct to L-DOPA for treating symptomatic deficits in motor movement and sensation and can protect DA neurons concomitantly, which may have critical implications in the neural pathways of motor and sensory disorders in PD.
