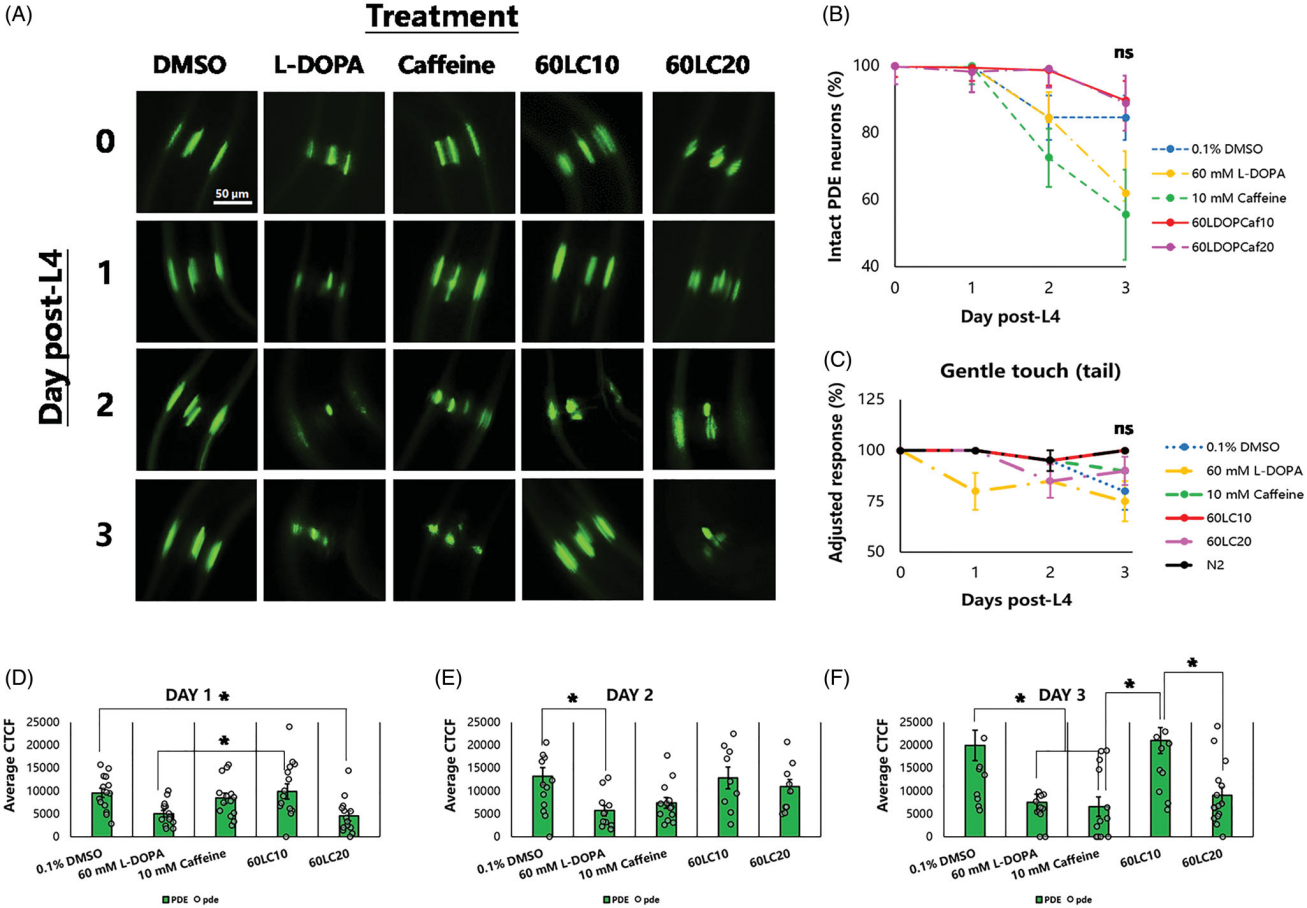# Correction

**DOI:** 10.1080/13880209.2020.1804676

**Published:** 2020-08-28

**Article title:** Caffeine reduces deficits in mechanosensation and locomotion induced by L-DOPA and protects dopaminergic neurons in a transgenic *Caenorhabditis elegans* model of Parkinson’s disease

**Authors:** Manalo, R. V. M., & Medina, P. M. B.

**Journal:** *Pharmaceutical Biology*

**Bibliometrics:** Volume 58, Number 1, pages 721–731

**DOI:** http://doi.org/10.1080/13880209.2020.1791192

Figure 3E was incorrectly replaced by a re-pasted Figure 3D instead of its original intended figure.